# Relation between patterns of intrinsic network connectivity, cognitive functioning, and symptom presentation in trauma‐exposed patients with major depressive disorder

**DOI:** 10.1002/brb3.664

**Published:** 2017-03-31

**Authors:** Melissa Parlar, Maria Densmore, Geoffrey B. Hall, Paul A. Frewen, Ruth A. Lanius, Margaret C. McKinnon

**Affiliations:** ^1^ McMaster Integrative Neuroscience Discovery and StudyMcMaster UniversityHamiltonONCanada; ^2^ Mood Disorders ProgramSt. Joseph's HealthcareHamiltonONCanada; ^3^ Department of PsychiatryUniversity of Western OntarioLondonONCanada; ^4^ Department of Psychology, Neuroscience, and BehaviourMcMaster UniversityHamiltonONCanada; ^5^ Homewood Research InstituteGuelphONCanada

**Keywords:** central executive network, cognition, default mode network, dissociation, fMRI functional connectivity analysis, major depressive disorder, salience network, trauma

## Abstract

**Objective:**

The present study investigated resting fMRI connectivity within the default mode (DMN), salience (SN), and central executive (CEN) networks in relation to neurocognitive performance and symptom severity in trauma‐exposed patients with major depressive disorder (MDD).

**Method:**

Group independent component analysis was conducted among patients with MDD (*n* = 21), examining DMN, SN, and CEN connectivity in relation to neurocognitive performance and symptom severity. Activation in these networks was also compared between the patient group and healthy controls (*n* = 20).

**Results:**

Among the patient group, higher levels of performance on measures of verbal memory and executive functioning were related to increased connectivity within the DMN (i.e., inferior parietal lobe; precuneus). Greater depression severity was related to reduced connectivity between the SN and a node of the DMN (i.e., posterior cingulate cortex) and higher depersonalization symptoms were related to enhanced connectivity between the SN and a node of the DMN (i.e., middle temporal gyrus). Higher symptoms of depersonalization were also associated with reduced integration of the DMN with the medial frontal gyrus. Relative to controls, patients with MDD showed greater connectivity of the ventromedial prefrontal cortex within the DMN.

**Conclusion:**

Intrinsic connectivity network patterns are related to cognitive performance and symptom presentation among trauma‐exposed patients with MDD.

## 1 Introduction

In addition to its core affective components, major depressive disorder (MDD) is characterized by dysfunction in frontal‐temporally mediated cognitive domains, including executive functioning (Snyder, 2012), processing speed (McDermott & Ebmeier, 2009), working memory (Gałecki et al., 2013), and recollective memory (Talarowska et al., 2010). These deficits are of significant concern as they contribute to impairments in multiple psychosocial domains (McIntyre et al., 2013) and are related to disruptions in instrumental activities of daily living (McCall & Dunn, 2003). Notably, patients with trauma‐related disorders, including post‐traumatic stress disorder (PTSD), show deficits in a similar range of cognitive domains (Cohen et al., 2013), with structural and functional brain changes observed across MDD and trauma‐related disorders in regions linked to recollective memory (e.g., hippocampus) (McKinnon, Yucel, Nazarov, & MacQueen, 2009), attention (e.g., anterior cingulate cortex [ACC]) (Yucel et al., 2008), and executive functioning (e.g., dorsolateral prefrontal cortex [dlPFC]) (Koenigs & Grafman, 2009). Critically, many patients with MDD have a comorbid history of trauma exposure, where in a sample of two thousand participants with anxiety and/or depressive disorders, only 8.8% failed to report experiencing a potentially traumatic or bothersome life event (Spinhoven, Penninx, van Hemert, de Rooij, & Elzinga, 2014). Trauma exposure impacts negatively on cognitive functioning (Olff, Polak, Witteveen, & Denys, 2014) and treatment outcome (Harkness, Bagby, & Kennedy, 2012) in patients with MDD and thus represents a critical variable to investigate in the context of depression. Here, we implement a symptom‐driven approach to examine the relation between patterns of intrinsic neural connectivity, cognitive functioning and symptom presentation (e.g., dissociation) among patients with a primary diagnosis of MDD and a history of trauma exposure.

Menon (2011) proposes a triple network model of psychopathology comprising three main intrinsic connectivity networks (ICNs) that together may underlie many of the neurocognitive and affective symptoms that characterize psychiatric disorders. This model describes the default mode network (DMN), the central executive network (CEN), and the salience network (SN). The DMN is defined most consistently by cortical midline structures (posterior cingulate cortex [PCC]: BA 23 and 31, ventromedial prefrontal cortex) (Buckner, Andrews‐Hanna, & Schacter, 2008), and is involved in self‐related processes, social cognition, and autobiographical memory. This network shows reductions in activation during the performance of cognitively demanding tasks (Greicius, Krasnow, Reiss, & Menon, 2003). By contrast, the CEN, consisting of the dlPFC and posterior parietal cortex, is instrumental in executive functioning and is engaged during cognitively demanding tasks requiring attention (Seeley et al., 2007). Finally, the SN is comprised of the ventrolateral prefrontal cortex, the anterior insula, and the dorsal anterior cingulate cortex (dACC) (Seeley et al., 2007) and plays a role in salience detection. The SN, in particular the anterior insula, is thought to be partly responsible for switching between the DMN and the CEN, thus facilitating the engagement of brain regions mediating higher‐order cognitive processes (Menon, 2011; Sridharan, Levitin, & Menon, 2008). By examining the association between ICNs, cognitive dysfunction and symptom presentation in patients with MDD who have a history of trauma exposure, we hope to increase our understanding of the neural mechanisms underlying cognitive dysfunction and symptom presentation among a patient population that is often treatment resistant and presents with a heightened pattern of cognitive dysfunction (Harkness et al., 2012; Olff et al., 2014).

Resting brain activity is related to behavioral performance on cognitive tasks in various populations, where preliminary results suggest that elevated connectivity within the DMN and within the CEN is related to enhanced performance on cognitive tasks. For example, connectivity within the DMN is positively associated with verbal working memory among healthy adults (Yakushev et al., 2013), and with memory performance (using a memory composite score) among older adults (Ward et al., 2014). Healthy controls also demonstrated an association between CEN connectivity and superior performance on Trails B, a measure of alternating attention (Seeley et al., 2007). In related studies, patients with Parkinson's disease show elevated DMN connectivity in association with faster processing speed, better executive functioning (Disbrow et al., 2014), and higher memory and visuospatial task scores (Tessitore et al., 2012). Heightened functional connectivity of executive control networks during rest was also related to a reduced Stroop effect (i.e., better executive control) among participants with Internet gaming disorder and healthy controls (Dong, Lin, & Potenza, 2015).

Only recently has the association between cognitive performance and resting‐state connectivity been explored in patients with depression. For example, performance on a list‐learning task was positively associated with increased connectivity of the parahippocampus with the amygdala and other subcortical structures, regions often showing weaker connectivity in MDD (Rao et al., 2016).

Resting‐state fMRI studies in patients with depression have consistently identified abnormalities across ICNs, suggesting a reorganization of intrinsic functional connectivity that could contribute to symptoms of MDD (Manoliu et al., 2013). The majority of these studies have focused on the DMN, reporting that patients with MDD tend to show abnormal activation during goal‐directed tasks, and increased functional connectivity during rest (for a review, see Broyd et al. (2009)). Overall, it appears that patients with MDD have difficulty downregulating activity within the DMN, which may account for symptoms such as increased rumination (Nejad, Fossati, & Lemogne, 2013) and impaired attentional control (Marchetti, Koster, Sonuga‐Barke, & De Raedt, 2012). Moreover, unmedicated adults with MDD show increased connectivity of the DMN with the SN (Greicius et al., 2007). Additional studies, however, found reduced integration of the anterior and posterior DMN in patients with depression (de Kwaasteniet et al., 2015; Sambataro, Wolf, Pennuto, Vasic, & Wolf, 2013).

Aberrant connectivity of the CEN has also been observed during task performance (Fitzgerald et al., 2008) and at rest (Diener et al., 2012) in patients with MDD. Interestingly, increased activation of the anterior insula in response to negative stimuli has been shown in MDD (Strigo et al., 2008), and increased connectivity of the dorsal mid‐insula cortex with limbic areas is positively correlated with depressive symptom severity (Avery et al., 2014) (but see Alexopoulos et al. (2012)). The right fronto‐insular cortical network, however, was found to be less active at rest in patients with MDD as compared to controls (Hamilton et al., 2012). Finally, remitted youth with MDD demonstrate hyperconnectivity of the DMN and SN with the cognitive control network (CCN), as compared to controls (Jacobs et al., 2014).

Despite the high prevalence of trauma exposure in depression and its negative effects on cognitive functioning and treatment outcome, no studies have examined alterations in ICNs in relation to cognitive functioning and symptom presentation, including dissociation, in this patient population. This effort may assist in better understanding the neural mechanisms underlying the reduced treatment and functional outcomes observed in this population. Critically, trauma exposure has been shown to lead to changes in ICNs, even without the development of PTSD (Kennis, Rademaker, Van Rooij, Kahn, & Geuze, 2015; Wang et al., 2014). In addition, scores on the Dissociative Experiences Scale (indexing dissociative symptoms of PTSD including depersonalization and derealization) positively correlated with DMN connectivity with the dlPFC (a node of the CEN) in patients with PTSD related to early life trauma (Bluhm et al., 2009). Moreover, dissociative symptoms have been related to decreased integration of anterior and posterior DMN components (Tursich et al., 2015). Despite the increasing focus on dissociative symptoms in MDD and their relation to more severe symptoms and trauma exposure (Molina‐Serrano, Linotte, Amat, Souery, & Barreto, 2008; Žikić, Ćirić, & Mitković, 2009), it is unknown whether these symptoms are associated with altered network connectivity in patients with depression and a history of trauma.

In this study, we investigate those core neural networks thought to be central to higher‐order cognitive functioning to determine if alterations in the connectivity of ICNs is related to neurocognitive performance and symptom presentation among trauma‐exposed patients with MDD. To our knowledge, there are only three studies that examine the relation between cognitive functioning and ICNs in patients with MDD, including Rao et al.'s (2016) study on list learning and resting‐state connectivity, as mentioned earlier. Jacobs et al. (2014) studied youth with remitted MDD and found that hyperconnectivity of the DMN and SN with the CCN was positively correlated with greater sustained attention on a Go/No‐Go task. In a sample of patients with late‐life depression, low resting functional connectivity within the CCN prior to treatment was related to greater dysexecutive behavior after treatment (Alexopoulos et al., 2012). To date, however, no studies have examined the relation between these core neural networks and performance on standardized neuropsychological assessments tapping frontal‐temporally mediated cognitive domains in patients with MDD and a history of trauma.

### 1.1 Aims of the study

Here, we examine patterns of intranetwork connectivity of the DMN, SN, and CEN in patients with MDD and a history of trauma relative to controls via independent components analysis (ICA). We also examine the association between scores on standardized neuropsychological assessments and measures of symptom severity (e.g., dissociation) and intranetwork connectivity within the patient group, across all three ICNs.

## 2 Material and Methods

### 2.1 Participants

This study was approved by the Hamilton Integrated Research Ethics Board of McMaster University and St. Joseph's Healthcare, Hamilton. Twenty‐one right‐handed patients (mean age: 40.2 (14.9), 10 males, 11 females) who met DSM‐IV diagnostic criteria for a primary diagnosis of recurrent (i.e., ≥3 episodes) MDD on the Structured Clinical Interview for DSM‐IV‐TR Axis I Disorders (SCID‐1; First, Spitzer, Gibbon, & Williams, 1997) and who had a history of trauma exposure, according to responses on the Clinician‐Administered PTSD Scale (CAPS; Blake et al., 1995) and/or Childhood Trauma Questionnaire (CTQ; Bernstein et al., 2003) were recruited. Among patients with MDD, five met criteria for moderate‐to‐severe childhood abuse on the CTQ only, nine met criteria for lifetime PTSD or trauma exposure on the CAPS only, and seven met criteria for childhood trauma exposure on the CTQ and a diagnosis of PTSD or trauma exposure on the CAPS. Among those participants who met criteria for PTSD or trauma exposure on the CAPS, 11 experienced interpersonal trauma (e.g., abuse by caregiver) and five experienced single‐blow, accidental trauma (e.g., car accident). Five participants met criteria for PTSD at time of testing. A control group consisted of 20 right‐handed participants with no history of psychiatric illness or trauma exposure who did not differ from the patient group in age, gender, or education (mean age: 35.3 (14.1), 10 males, 10 females) (see Table 1 for demographic and clinical characteristics).

**Table 1:** Demographic and clinical characteristics of study sample

Characteristic	MDD (*n* = 21)	Controls (*n* = 20)
	Mean (*SD*)	Mean (*SD*)
Demographic characteristics		
Age	40.2 (14.9)	35.3 (14.1)
Years of education	15.5 (3.7)	17.2 (2.7)
Gender (female:male)	11:10	10:10
Ethnicity (Caucasian) frequency	19	17
Employment status (employed/unemployed) frequency	8^a^/13	14/6
Clinical characteristics		
HAM‐D	12.0^b^ (5.8)	0.5 (0.8)
CAPS (month)	29.1^b^ (31.2)	0.0 (0.0)
MDI		
Depersonalization	7.4^b^ (2.6)	5.1 (0.2)
Derealization	7.7^b^ (3.6)	5.3 (0.6)
Sum (depersonalization + derealization)	15.1^b^ (5.6)	10.4 (0.6)
CTQ		
Total	48.7^b^ (15.9)	30.6 (3.7)
Emotional abuse	13.0^b^ (5.8)	6.5 (1.3)
Physical abuse	7.8^b^ (3.2)	5.5 (0.8)
Sexual abuse	7.3^b^ (5.3)	5.0 (0.0)
Emotional neglect	13.2^b^ (4.7)	8.1 (3.0)
Physical neglect	7.5^b^ (1.9)	5.6 (0.8)
Number of depressive episodes	13.2 (15.6)	0.0 (0.0)
Age onset of depression	19.8 (9.9)	N/A

CAPS, Clinician‐Administered PTSD Scale; CTQ, Childhood Trauma Questionnaire; HAM‐D, Hamilton Depression Rating Scale; MDD, major depressive disorder; MDI, Multiscale Dissociation Inventory.

a Significant group effect (*p *<* *.05).

b Significant group effect (*p *<* *.01).

Exclusion criteria included past or current diagnosis of bipolar disorder, a psychotic disorder, neurological disease, electroconvulsive or transcranial magnetic stimulation therapy in the last 12 months, any contraindication to MRI, traumatic brain injury and/or head injury with loss of consciousness (lasting more than 60 s), substance abuse in the last 6 months, current or lifetime history of substance dependence, and/or current or prior history of untreated significant medical illness. Participants were instructed not to use benzodiazepines within 12 hrs prior to testing.

### 2.2 Measures

#### 2.2.1 Clinical assessments

In addition to the SCID‐I, participants completed the 17‐Item Hamilton Rating Scale for Depression (HAM‐D; Hamilton, 1960). This interview assesses the severity of depressive symptoms over the past week. The CAPS (Blake et al., 1995) was also administered to assess current (i.e., past month) and past PTSD diagnostic status and symptom severity and to confirm a history of trauma exposure. Participants completed the CTQ (Bernstein et al., 2003), a 28‐item self‐report questionnaire that measures history of: (1) emotional abuse, (2) physical abuse, (3) sexual abuse, (4) emotional neglect, and (5) physical neglect. The CTQ has good internal consistency and convergent reliability with therapists' assessments of history of trauma is high (Bernstein et al., 2003). Participants also completed the Multiscale Dissociation Inventory (MDI; Briere, 2002) a 30‐item self‐report questionnaire that assesses dissociative responses. The current study focused on MDI depersonalization and derealization subscales, as well as a composite score of the sum of these two subscales, as these symptoms have been associated with increased depressive symptomatology (Žikić et al., 2009) and form the basis of the recently described dissociative subtype of PTSD (Spiegel et al., 2013).

#### 2.2.2 Neuropsychological assessment battery

The following battery of standardized neuropsychological measures was administered to assess frontal‐temporally mediated domains of cognitive functioning: *Declarative memory*: (1) California Verbal Learning Test—Second Edition (standard form) (CVLT‐II; Delis, Kramer, Kaplan, & Ober, 2000) a word learning task that provides indices of immediate and delayed memory performance and recognition. *Executive functioning*: (1) Controlled Oral Word Association Test (COWAT): assesses phonemic (F, A, S) fluency (Gladsjo et al., 1999); (2) Color Trail Making Test (Parts 1 and 2) (D'Elia, Satz, Uchiyama, & White, 1996): measures attention, speed, and mental flexibility; (3) Wisconsin Card Sorting Test (128‐item version) (WCST; Heaton, 2003): measures the ability to form and shift concepts based on feedback. *Attention*: (1) Conners' Continuous Performance Test—Second Edition (CPT‐II; Conners, 2000): a computerized task assessing sustained attention, response inhibition, and impulsivity.

#### 2.2.3 Imaging paradigm and acquisition

Participants underwent a 4‐min eyes‐open resting‐state scan during which they were shown a black fixation cross on a gray screen to ensure greater reliability of within network connectivity as compared to eyes‐closed conditions (Patriat et al., 2013), following standard procedures (Ros et al., 2013). All images were obtained using a 3.0 Tesla whole‐body MRI scanner (General Electric, Milwaukee, WI, USA) using an 8‐channel parallel receiver birdcase head coil (General Electric). For the resting state task, 34 axial slices (3 mm thick, no skip) across the whole brain were imaged using a gradient echo pulse sequence (echo time [TE] = 35 ms; repetition time [TR] = 3000 ms; acquisition matrix = 64 × 64; FOV = 24 cm; flip angle = 90°) to obtain a total of 80 volumes per participant. A TR of 3,000 ms was chosen to ensure enough slices per volume were acquired to obtain a full coverage of the brain and to minimize the risk of losing data due to larger head sizes of some participants.

### 2.3 Statistical analyses

#### 2.3.1 Demographics, psychological, and cognitive characteristics

To assess group differences in demographic, clinical, and cognitive variables, data were first assessed for normality (*p* > .05, Shapiro**–**Wilk) and group comparisons were calculated using either independent‐samples *t* tests or Mann**–**Whitney *U* tests, depending on distribution of data. Group differences in gender and employment status were assessed with chi‐square tests. Analysis of demographic, psychological, and cognitive data was performed in IBM SPSS Version 22.0.

#### 2.3.2 Image preprocessing

Preprocessing of images (slice‐timing correction, motion correction, spatial normalization, and smoothing) was performed using SPM8 (RRID:SCR_007037) (http://www.fil.ion.ucl.ac.uk/spm/) in MATLAB 8.3.0 (MathWorks Inc.) using standard procedures. Individual functional images were corrected for motion by realignment to the first volume of each session, resliced, and a mean functional volume created. The mean image was coregistered to the standard echo‐planar imaging template in MNI space supplied by SPM8, the deformation matrix was then created and applied to the functional volumes. All functional volumes were smoothed using a full‐width half‐maximum Gaussian filter of 8 mm.

#### 2.3.3 Functional connectivity analysis

We performed ICA (Calhoun, Kiehl, & Pearlson, 2008; Calhoun, Liu, & Adali, 2009) of the resting‐state functional data using the Group ICA of fMRI Toolbox (GIFT) (RRID:SCR_001953) (GROUPICAT v3.0a/GIFT v2.0a; http://mialab.mrn.org) to identify ICNs for all 41 participants. We isolated 20 ICs using the Infomax algorithm in GIFT, as this number of components shows the strongest correspondence with region‐of‐interest (ROI) data (Rosazza, Minati, Ghielmetti., Mandelli, & Bruzzone, 2012), and repeated the estimation 20 times using the ICASSO method (Himberg, Hyvärinen, & Esposito, 2004) to enhance component reliability. Single subject spatial maps and time courses for each component were back‐reconstructed for each participant and converted to *z*‐scores to indicate the strength of each voxel's contribution to the component (Erhardt et al., 2011). The spatial sorting function in GIFT was used to identify the components whose spatial pattern showed the highest correlation (Pearson's *r*) with network templates (DMN and CEN available from http://findlab.stanford.edu/functional_ROIs.html; Shirer, Ryali, Rykhlevskaia, Menon, & Greicius, 2012) (RRID:SCR_014757) and with network masks from our previous studies (SN; Laird et al., 2009; Lanius, Frewen, Tursich, Jetly, & Mckinnon, 2015). Components of artifact signal and head movement did not correlate with the DMN, SN, or CEN network masks and were therefore not incorporated into group analyses. The Artifact Detection Tool (ART) software package (https://www.nitrc.org/projects/artifact_detect/) was used to further ensure groups did not differ in their movement parameters. Specifically, we coded whether the ART software identified any outliers within each individual scan, and carried out a Fisher's exact test to examine group differences. This analysis indicated that groups did not differ in movement parameters (*p* = .75).

#### 2.3.4 Statistical comparison of spatial maps

Component *z*‐score maps of interest were imported into SPM8 for group analysis. ROI analyses were carried out through the a priori defined standardized masks of the DMN (Shirer et al., 2012), CEN (Shirer et al., 2012) and SN (Kluetsch et al., 2014; Ros et al., 2013). Cluster‐level significance was denoted by *p*FDRc (false discovery rate), and voxel‐level significance was denoted by *p*FDRv. Second‐level multiple regression analyses were performed in SPM8 to determine the association between the components of interest and (1) neuropsychological test scores (raw) and (2) clinical variables (i.e., MDI depersonalization, derealization, and composite scores, and HAM‐D total) among the trauma‐exposed group with MDD. ROI analyses were again carried out and thresholded at *p*FDR < .05 to control for multiple comparisons using the a priori defined standardized masks.

## 3 Results

### 3.1 Participants

Groups did not differ in terms of age, years of education, gender distribution, or ethnicity. The groups differed with respect to employment status, where the trauma‐exposed MDD group had a significantly higher proportion of unemployed participants (χ^2^_(1,40)_ = 4.19, *p *<* *.05). The trauma‐exposed MDD group scored significantly higher than the control group on all clinical subscales. See Table 1 for a description of the clinical and demographic characteristics of the study sample.

### 3.2 Neuropsychological performance

Compared to controls, participants with MDD and comorbid trauma performed significantly worse on measures of verbal memory and executive functioning (i.e., mental flexibility, abstract reasoning, and forming concepts) (see Table 2). Specifically, patients retrieved significantly fewer words on the CVLT‐II long‐delay free recall subscale (*t* (39) = 2.1, *p *<* *.05) as compared to controls. On Color Trails 2, patients were significantly slower than controls (*U *=* *90.0, *p *<* *.01). Patients also made more total errors on the WCST than controls, however, these results did not reach statistical significance (*U *=* *117.5, *p *=* *.07). No group differences emerged on the COWAT phonemic fluency, CPT‐II number of commissions or omissions, Color Trails 1, CVLT‐II total and short‐delay free recall, or WCST perseverative error raw scores.

**Table 2:** Neuropsychological performance of patients with MDD and trauma and healthy controls

Characteristic	MDD (*n* = 21)	Controls (*n* = 20)
	Mean (*SD*)	Mean (*SD*)
Measure		
CVLT‐II total raw	51.6 (9.2)	56.0 (9.8)
CVLT‐II short‐delay free recall raw	11.4 (2.6)	12.5 (3.3)
CVLT‐II long‐delay free recall raw	11.4^b^ (2.5)	13.2 (2.8)
COWAT phonemic (FAS)	46.3 (11.8)	42.8 (12.1)
Color Trails 1 raw (sec)	31.4 (13.3)	26.8 (7.8)
Color Trails 2 raw (sec)	67.1^c^ (20.8)	51.0 (13.9)
WCST total errors raw	23.5^a^ (18.5)	19.9 (20.2)
WCST perseverative errors raw	12.1 (11.8)	11.1 (11.1)
CPT‐II omissions raw	4.2 (12.7)	1.6 (3.0)
CPT‐II commissions raw	13.6 (7.5)	11.7 (5.4)

CVLT‐II, California Verbal Learning Test‐II; COWAT, Controlled‐Oral Word Association Test; MDD, major depressive disorder; CPT‐II, Conners' Continuous Performance Test; WASI, Wechsler Abbreviated Scale of Intelligence; WCST, Wisconsin Card Sorting Test.

a Trending group effect (*p *=* *.07).

b Significant group effect (*p *<* *.05).

c Significant group effect (*p *<* *.01).

### 3.3 Component identification

**Figure 1:**
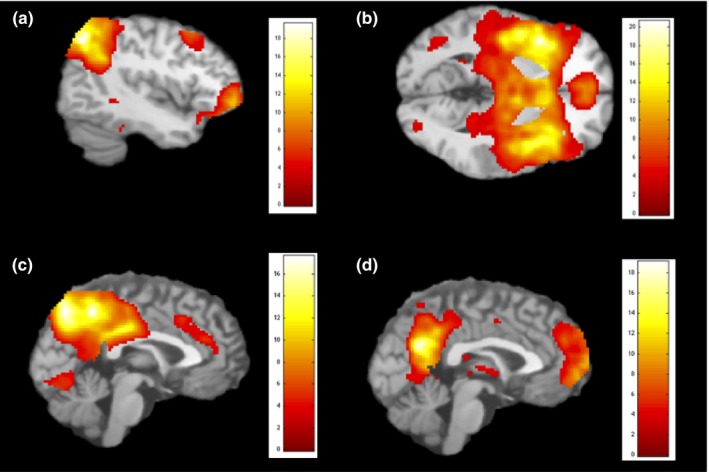
Independent components (IC) extracted for: (a) the central executive network (CEN), IC 8; (b) the salience network (SN), IC 16; (c) the default mode network (DMN), IC 9; (d) the default mode network (DMN), IC 18

Four artifact‐free components showed moderate‐to‐high spatial correlations with the predefined masks of the CEN (IC 8, *r = *.46), SN (IC 16, *r = *.52), and the DMN (IC 9, *r = *.64, and IC 18, *r = *.38) (see Figure 1). IC 8 (CEN) encompassed a large area of the right dlPFC (including the superior [BA 8] and inferior prefrontal cortex [BA 47]), extending to the bilateral inferior (BA 40) and superior (BA 7) parietal lobes, and left middle temporal gyrus (MTG) (BA20, 21). IC 16 (SN) covered the left insula (BA 13), extending to bilateral inferior frontal gyrus (BA 47), dorsal ACC and ventral ACC, as well as mPFC. IC 9 (DMN) consisted of the precuneus (BA 7), and ACC (BA 24, 32). IC 18 (DMN) encompassed the PCC, the right MTG (BA 39), medial prefrontal cortex (BA 9, 10), and the right inferior parietal lobe (IPL) (BA 40). IC 8, IC 16, and IC 9/18 will be referred to as CEN, SN, and DMN respectively.

### 3.4 Statistical comparison of spatial maps

**Figure 2:**
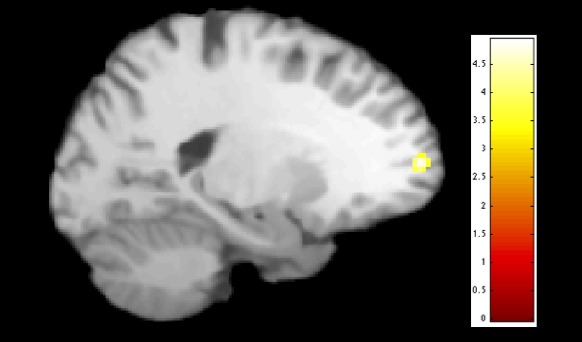
Between group differences within the default mode network: compared to healthy controls, patients with major depressive disorder (MDD) showed a higher integration of the vmPFC (*p* = .048, FDR‐corrected, *t* = 4.93) within IC 9

Compared to controls, participants with depression had increased functional integration of the left ventromedial PFC (vmPFC) within the DMN (IC9) (MNI coordinates: −18, 56, 8; *t *=* *4.93, *p*FDRc = .048, BA10) (see Figure 2). No differential connectivity was found within the SN and CEN between the two groups.

### 3.5 Cognitive performance and spatial distribution

**Figure 3:**
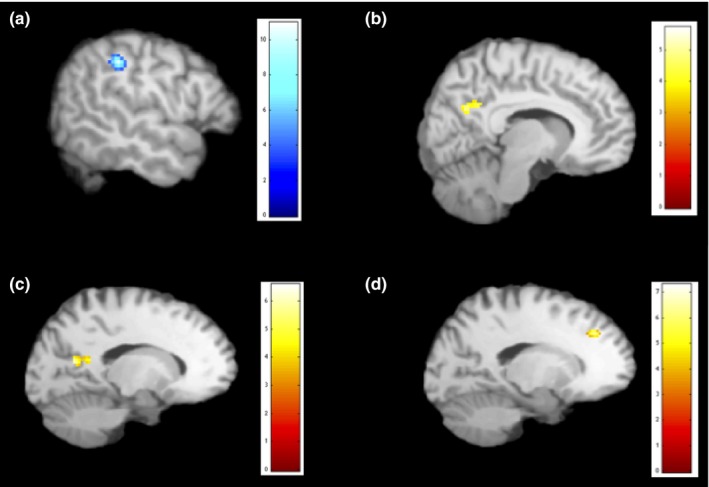
Regions (in blue) displaying positive correlations and regions (in yellow) displaying negative correlations with cognitive and clinical varibales. (a) correlation between CVLT‐II LDFR and integration of the IPL within the DMN; (b) correlation between completion time on Color Trail Making Test—Part 2 and integration of the precuneus within the DMN; (c) correlation between HAM‐D scores and connectivity of the PCC within the SN; (d) correlation between MDI depersonalization scores and integration of the medial frontal gyrus within the DMN. CVLT‐II LDFR, California Verbal Learning Test‐II Long‐Delay Free Recall; DMN, default mode network; HAM‐D, Hamilton Rating Scale for Depression; IPL, inferior parietal lobe; MDI, Multiscale Dissociation Inventory; PCC, posterior cingulate cortex; SN, salience network

Second‐level multiple regression analyses were performed among the group of trauma‐exposed participants with MDD using their *z*‐score spatial maps to examine the associations between cognitive performance variables and the CEN, SN, and DMN. Within the SN, better performance on the Color Trail Making Test—Part 1 (i.e., faster completion time), was associated with increased connectivity of the left superior temporal gyrus (STG) (MNI coordinates: −36, −2, −20, *t *=* *5.20, *p*FDRv = .049, BA38). Within the DMN (IC 18), there was a positive correlation between CVLT‐II long‐delay free recall scores and the integration of the left IPL (MNI coordinates: −50, −36, 36, *t *=* *10.95, *p*FDRv < .001, BA40) (see Figure 3a). Furthermore, increased connectivity of the right middle occipital gyrus within the DMN was related to worse performance on COWAT phonemic fluency (MNI coordinates: 52, −58, −8, *t *=* *5.65, *p*FDRc = .037, BA19). Finally, increased integration of the precuneus within the DMN was related to faster completion time on the Color Trail Making Test—Part 2 (MNI coordinates: 14, −54, 28, *t *=* *5.72, *p*FDRc = .015, BA31) (see Figure 3b). The CEN did not show any significant associations with cognitive test scores. Scores on the CPT‐II and WCST were not significantly correlated with any of the components.

### 3.6 Clinical symptoms and spatial distribution

Higher HAM‐D scores were associated with reduced connectivity of the PCC within the SN (MNI coordinates: −10, −50, 14, *t* = 6.57, *p*FDRc = .006, BA29) (Figure 3c). Moreover, within the SN, increased integration of the right MTG was related to higher scores on the MDI composite score (MNI coordinates: 52, −62, 8, *t* = 7.17, *p*FDRv = .055, BA39). Within the DMN, increased integration of the medial frontal gyrus was related to lower scores on the MDI depersonalization subscale (MNI coordinates: −14, 34, 36, *t* = 7.29, *p*FDRv = .045, BA9) (Figure 3d).

## 4 Discussion

To our knowledge, this is the first study to examine the functional connectivity of the CEN, SN, and DMN in relation to cognitive performance and clinical symptoms in trauma‐exposed patients with MDD. We found increased integration of the vmPFC within the DMN in patients compared to controls. Moreover, among the patient group, cognitive performance variables and symptoms of depression and dissociation were associated with distinct connectivity patterns within the three ICNs of interest. Specifically, greater connectivity of the SN with the STG was associated with better performance on an attention task, whereas greater connectivity of the DMN with the IPL and precuneus was related to higher levels of performance on tasks of verbal memory and executive functioning, respectively. Whereas greater severity of depressive symptoms was related to reduced connectivity of the PCC within the SN, higher dissociation scores were associated with increased connectivity of the right MTG within the SN, and decreased integration of the medial frontal gyrus within the DMN.

Comparing ICN activation patterns between patients with depression and controls, the only significant group difference that emerged was within the DMN, where patients demonstrated enhanced integration of the vmPFC within the DMN, as compared to controls. The vmPFC not only represents a core region of the DMN (Buckner et al., 2008), but it is also a critical region involved in self‐referential processing (Lemogne, Delaveau, Freton, Guionnet, & Fossati, 2012; Northoff et al., 2006). This finding suggests hyperconnectivity within the DMN in patients with MDD, and is consistent with previous studies in patients with depression (Alexopoulos et al., 2012; Kaiser, Andrews‐Hanna, Wager, & Pizzagalli, 2015; Sheline, Price, Yan, & Mintun, 2010) (but see de Kwaasteniet et al. (2015) and Zhu et al. (2012), for conflicting findings). Among patients with PTSD, however, it is more common to observe disruptions in DMN integration (Bluhm et al., 2009; Rabellino et al., 2015; Sripada et al., 2012) where it has been proposed that early‐life trauma disrupts the integration of the DMN (Daniels et al., 2010). Despite having a history of trauma exposure, the primary diagnosis among our patient sample was MDD, and the timing, type, and frequency of trauma were heterogenous among participants. These characteristics of our patient group may account for the increased connectivity observed within the DMN, compared to the pattern commonly observed in patients with PTSD. Indeed, future studies may compare patients with MDD with and without childhood trauma to explore if early‐life trauma in this population results in reduced integration within the DMN. The finding that the vmPFC in particular showed enhanced connectivity in our patient group is particularly relevant given the role of the vmPFC in depressive self‐referential focus (Lemogne et al., 2012). Clinically, this may manifest as increased rumination during the resting‐state task in our patient group.

We found significant correlations between cognitive variables and connectivity within both the SN and DMN among the trauma‐exposed participants with MDD. Better performance on the Color Trail Making Test—Part 1, a measure of attention and processing speed, was related to greater connectivity of the left STG within the SN. The role of the STG in cognitive tasks has been demonstrated in patients with multiple sclerosis (Achiron et al., 2012) and healthy controls (Bayless, Gaetz, Cheyne, & Taylor, 2006; Paulus, Feinstein, Leland, & Simmons, 2005). Specifically, the STG is activated during cognitive tasks requiring decision‐making and thickness of the left STG correlates with information processing speed on the Mindstreams Computerized Cognitive Battery (Achiron et al., 2012). Given the role of the STG in cognitive processing, our results suggest that increased functional connectivity of the SN with this region may be a protective factor against impairments in attention and processing speed among patients with MDD. By contrast, decreased connectivity would be expected to result in cognitive processing impairments.

Within the DMN, increased connectivity with the left IPL was related to better performance on the CVLT‐II long‐delay free recall condition, measuring delayed verbal memory. Notably, patients performed significantly worse than controls in the CVLT‐II long‐delay free recall condition. The posterior parietal cortex is involved in attention and episodic memory retrieval, and is activated during verbal memory tasks (Hutchinson, Uncapher, & Wagner, 2009). Moreover, the IPL (BA 40) is considered to be a core component of the DMN (Buckner et al., 2008). As previously noted, patients with depression tend to show increased connectivity within the DMN as compared to controls, which is often viewed as reflecting increased self‐referential focus and difficulty disengaging the task‐negative state when needed. In our sample, however, increased integration of the DMN with a core region of this network was related to better neuropsychological performance. Interestingly, studies in other populations, such as in patients with Parkinson's disease (Disbrow et al., 2014; Tessitore et al., 2012), healthy older adults (Ward et al., 2014), and healthy controls (Yakushev et al., 2013) have reported that greater connectivity within the DMN is associated with better performance on different memory tasks. Our results are also consistent with recent findings suggesting that among patients with depression, but not healthy controls, better performance on a delayed recall trial of list learning was predictive of greater connectivity between the parahippocampus and subcortical structures (Rao et al., 2016). Taken together, these results suggest that greater connectivity among regions supporting memory retrieval (e.g., IPL and parahippocampus) and other brain areas may be conducive to enhanced retrieval among patients with MDD.

Similarly, our results indicate that increased connectivity of the precuneus (a core component of the DMN) within the DMN was related to better performance on Color Trail Making Test—Part 2. This task measures processing speed, mental flexibility, and working memory, and compared to controls, the patient group was significantly slower at completing this task, reflecting poorer performance. Hence, consistent with our findings on the CVLT‐II and DMN connectivity, we again see increased integration of the DMN associated with superior performance on a cognitive task. Importantly, the precuneus is involved in mental processes and self‐operations (Cavanna & Trimble, 2006). Finally, within the DMN, increased integration of the middle occipital gyrus was related to worse performance on our measure of verbal fluency, the COWAT phonemic fluency subscale. The left middle occipital gyrus has been implicated in working memory processes in PTSD, where increased connectivity between this region and the mPFC was present during a working memory task (Daniels et al., 2010). Veer et al. (2010) found reduced connectivity of visual regions in patients with depression, but noted that this region is rarely implicated in depression and interpretations would therefore be speculative. Although our results suggest that increased connectivity of the DMN with this region is related to poorer verbal fluency, further research will be required to interpret these findings.

Significant correlations also emerged between clinical variables (i.e., HAM‐D and MDI) and each of the three ICNs. Within the SN, higher HAM‐D scores were associated with reduced connectivity of the PCC, suggesting that greater depression severity is related to decreased integration of the SN within a node of the DMN. These results are contrary to earlier studies in patients with MDD, where it was reported that depression was associated with an increased integration of the SN with the DMN (Greicius et al., 2007; Jacobs et al., 2014). A possible factor accounting for the discrepancy in findings could be the history of trauma‐exposure in our patient sample. For example, Wang and colleagues found that compared to patients with MDD without a history of trauma, those with trauma displayed a more widespread reduction in functional connectivity strength (Wang et al., 2014). Moreover, among patients with PTSD, abberant resting‐state connectivity between the DMN and SN has been observed (Bluhm et al., 2009; Lanius et al., 2010). As suggested by Daniels, Frewen, McKinnon, and Lanius (2011), early‐life trauma may impact negatively on the integration of the DMN, and thus could also impact inter‐network connectivity. Despite the heterogeneity among trauma subtype in our sample, the majority did report experiencing childhood trauma. Within the SN, increased integration of the right MTG, a component of the DMN (Laird et al., 2009), was related to higher scores on the MDI composite score (i.e., sum of depersonalization and derealization symptoms). The right MTG has been implicated in dissociative states, where patients with PTSD who dissociated during an fMRI traumatic script‐driven imagery paradigm demonstrated heightened levels of activation in this region, compared to controls (Lanius et al., 2002). This supports what has been termed the temporal lobe hypothesis of dissociation, based largely on epilepsy literature (Devinsky, Putnam, Grafman, Bromfield, & Theodore, 1989; Kenna & Sedman, 1965). On balance, these findings suggest that an enhanced connectivity of the right MTG within the SN, normally responsible for switching between the CEN and DMN, may underlie trait‐like dissociative symptoms in depression.

Depersonalization symptoms of dissociation, as assessed by the MDI, correlated negatively with connectivity of the medial frontal gyrus within the DMN. This region has been linked to self‐referential processing in depression (Lemogne et al., 2012). Interestingly, this finding suggests that higher levels of depersonalization (i.e., feeling as though one is disconnected from his/her body) are related to decreased connectivity of this core region associated with self‐referential processing. Similar findings have been reported in patients with PTSD (Tursich et al., 2015). Depersonalization is an important feature of depression, most often related to a history of comorbid trauma exposure and associated with greater illness severity (Molina‐Serrano et al., 2008; Žikić et al., 2009). These provocative findings suggest that alterations in the SN and DMN underlie dissociative symptoms in MDD, indicating an urgent need for future studies in order to address dissociative symptoms in patients with MDD and to better understand underlying neural mechanisms and their impact on treatment outcome.

The current study has several limitations. Most of the patients were taking psychotropic medications at time of testing (although they refrained from benozdiazepines for 12 hr prior to testing). There is a lack of knowledge and often inconsistent results surrounding the impact of antidepressants on resting state activity in MDD (Dutta, Mckie, & Deakin, 2014) and future studies should carefully control for medication status. Furthermore, we did not include a trauma‐exposed control group, or a nontrauma‐exposed MDD group. The sample size is small, yet similar to other neuroimaging studies examining resting‐state ICNs in psychiatric disorders. Unlike ROI analyses which rely on prior anatomic hypotheses, ICA is a data‐driven analysis. There are strengths and weaknesses inherent in both methods (Rosazza et al., 2012); therefore, future studies may wish to include network homogeneity and seed‐based functional connectivity analyses as alternate methods of examining network connectivity. In addition, we employed a 4‐minute resting state scan, which is sufficient to reliably estimate network connectivity. Given that increasing scan length can further improve reliability and reduce the risk of type II errors, longer resting conditions may be warranted (Van Dijk et al., 2010). In addition, we used a TR of 3,000 ms. This may be considered a limitation as larger TRs increase the risk of confounding physiological frequencies (Lowe, Mock, & Sorenson, 1998). Regarding our assessments, we measured symptoms of depression and dissociation, yet we did not assess state symptoms experienced during scanning or neuropsychological testing, which may have helped further elucidate the relation between these variables and ICNs. In addition, our assessments of trauma history were self‐report retrospective tools, which may present a memory bias.

To our knowledge, this is the first study to examine the association between neuropsychological performance and symptom presentation with resting‐state ICNs in a trauma‐exposed sample with MDD. This study is an important first step toward better understanding the neurobiological mechanisms underlying transdiagnostic features, such as cognitive dysfunction and dissociation, which are characteristic of depression and trauma‐related disorders. Importantly, our findings suggest that both cognitive performance and symptoms of depression and dissociation are related to DMN, SN, and CEN connectivity. Studies must continue to explore alterations in ICNs as a function of cognitive and clinical characteristics in this population with the goal of initiating treatment efforts that target altered functioning in these ICNs. Lanius et al. have proposed neuroscientifically informed treatment interventions to improve the functioning of the CEN, SN, and DMN in patients with PTSD, which may also have relevance to symptom domains of MDD (Lanius et al., 2015). Here, the authors proposed interventions such as top‐down cognitive remediation strategies, body scan meditations, and neurofeedback which may help to restore these neural networks and their related clinical and cognitive dysfunction. By increasing our understanding of the neural mechanisms underlying transdiagnostic variables characteristic of depression and trauma‐related disorders, we will be able to move forward and propose treatment efforts aimed at restoring these core networks altered by psychiatric illness.

## Conflict of Interest

None declared.
